# EpitopeViewer: a Java application for the visualization and analysis of immune epitopes in the Immune Epitope Database and Analysis Resource (IEDB)

**DOI:** 10.1186/1745-7580-3-3

**Published:** 2007-02-21

**Authors:** John E Beaver, Philip E Bourne, Julia V Ponomarenko

**Affiliations:** 1 San Diego Supercomputer Center, University of California, San Diego, 9500 Gilman Drive, La Jolla, California 92093, USA; 2 Department of Pharmacology, University of California, San Diego, 9500 Gilman Drive, La Jolla, California 92093, USA

## Abstract

**Background:**

Structural information about epitopes, particularly the three-dimensional (3D) structures of antigens in complex with immune receptors, presents a valuable source of data for immunology. This information is available in the Protein Data Bank (PDB) and provided in curated form by the Immune Epitope Database and Analysis Resource (IEDB). With continued growth in these data and the importance in understanding molecular level interactions of immunological interest there is a need for new specialized molecular visualization and analysis tools.

**Results:**

The EpitopeViewer is a platform-independent Java application for the visualization of the three-dimensional structure and sequence of epitopes and analyses of their interactions with antigen-specific receptors of the immune system (antibodies, T cell receptors and MHC molecules). The viewer renders both 3D views and two-dimensional plots of intermolecular interactions between the antigen and receptor(s) by reading curated data from the IEDB and/or calculated on-the-fly from atom coordinates from the PDB. The 3D views and associated interactions can be saved for future use and publication. The EpitopeViewer can be accessed from the IEDB Web site  through the quick link 'Browse Records by 3D Structure.'

**Conclusion:**

The EpitopeViewer is designed and been tested for use by immunologists with little or no training in molecular graphics. The EpitopeViewer can be launched from most popular Web browsers without user intervention. A Java Runtime Environment (RJE) 1.4.2 or higher is required.

## Background

The Immune Epitope Database and Analysis Resource (IEDB) aims to catalog and provide tools for the analysis of immune epitopes, defined by the IEDB as molecules recognized by immune receptors (antibodies, MHC molecules and T cell receptors) [1,2]. The three-dimensional (3D) structures of epitopes and antigens in complex with immune receptors are important components of the IEDB. The basic information on these molecular structures is available from the Protein Data Bank (PDB) [3]. As of February 2007 the PDB contained more than 700 structures of complexes of immunological interest. These raw data from the PDB are curated to provide epitope entries in the IEDB. Tools are needed to aid immunologists in fully understanding the molecular interactions of interest.

Current molecular visualization tools that enable the user to visualize and render biochemical structures include popular and freely available standalone applications that run on the desktop such as DeepView/Swiss-PdbViewer [4], RASMOL [5], Jmol [6], PyMol [7], BALLView [8], Cn3D [9], MDL Chime [10] and many others, including commercial viewers. Some of these viewers go beyond visualization and offer functionality for molecular modeling and simulation. For example, DeepView offers modeling, including amino acid mutation, energy minimization and homology modeling and BALLView includes molecular mechanics methods. Each of these tools requires the user to download and install an application on their client computer. Applications that can be launched directly from a Web browser include KiNG (Kinemage, Next Generation) [11], JmolApplet [6] and ProteinWorkshop from the PDB [12]. For more information about available molecular viewers one can visit the World Index of Molecular Visualization Resources web page [13]. One example of the resources listed here is the Online Macromolecular Museum [14] which provides visualization (with Chime) of structures of antibodies and MHC molecules. Another is the Antibody Resource [15], also developed using Chime. The associated browser-based SPICE viewer [16] displays annotations of proteins from PDB, UniProt and Ensembl. Finally the recently developed Conformational Epitope Database [17] and Epitome [18] provide conformational epitope visualization implemented using Jmol Java applets.

Web browser-based Java-applets have the advantage of ease of use – there is no application to download and install – but usually fail to deliver high-quality graphics and file export functionality provided by the most standalone viewers, for example, PyMol [7], Cn3D [9] and BALLView [8]. The availability of the Molecular Biology Toolkit (MBT) [19], with its use of 3D graphics libraries, makes possible the development of Java-applets offering high-quality graphics and export of publication-quality images. This has already been proven by the development of applications such as the Protein Kinase Resource viewer [20], Ligand Explorer (LigPro) [21] and ProteinWorkshop [12]. MBT provides a well-organized assortment of core classes that provide a uniform data model for the description of biological structures and automates common tasks associated with the development of applications in the molecular sciences, for example, data loading, derivation of typical structural information, visualization of sequence and standard structural entities [19].

Here, we present the EpitopeViewer, a visualization tool based on MBT and developed for visualization and analysis of 3D structures of immunological epitopes and their interaction with immune receptors. The EpitopeViewer is developed as a web browser-based Java-application and should run on any computer with Java-enabled (the default). The EpitopeViewer is specifically designed to work with data on structural epitopes curated and provided in the IEDB.

## Implementation Details and Features

The EpitopeViewer is implemented as a web browser-based Java application started using Java Web Start. Launching of the EpitopeViewer requires Java Runtime Environment 1.4.2 or higher be installed on the user PC. The software components necessary for the tool are downloaded and installed on the user PC automatically during the process of the viewer launching with the user's permission. All software components are freely available. As stated above, the EpitopeViewer is based on the Molecular Biology Toolkit [19], a set of foundation Java classes for visualizing a variety of biological data. The EpitopeViewer and MBT are written in Java and use JOGL (Java binding for OpenGL [22]). Even for the largest structure in the PDB [PDB:1HTQ] with 97,872 atoms, MBT loads in about a minute, requires about 350 MB of RAM, and is capable of rendering 0.5–6 frames per second (video card dependent). For an average structure in the PDB [e.g., PDB:1ATP], MBT requires about 60 MB of RAM, and renders approximately 20–120 frames per second (video card dependent). The MBT code works correctly with about 90% of current video cards tested.

**Figure 1:**
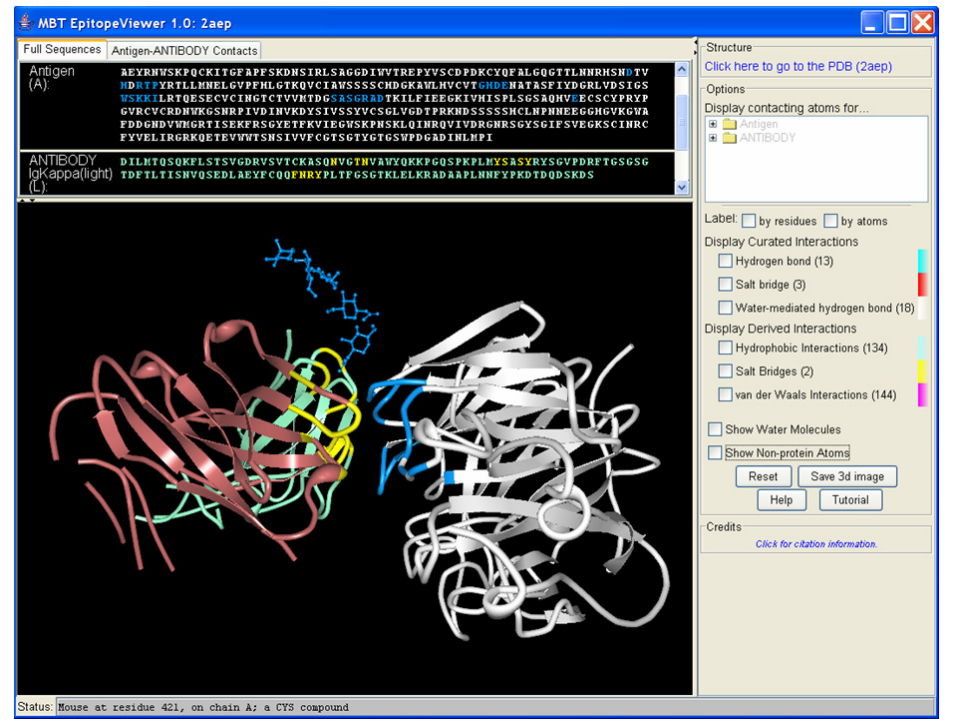
A screen-shot of the EpitopeViewer output represented the structure of neuraminidase recognized by Mem5 Fab [IEDB:1000520, PDB:2AEP] [27]. Neuraminidase is in white, light chain of the Fab is in bluish-green, heavy chain is in reddish-brown, epitope is in blue, antibody residues interacting with antigen are in yellow.

**Figure 2:**
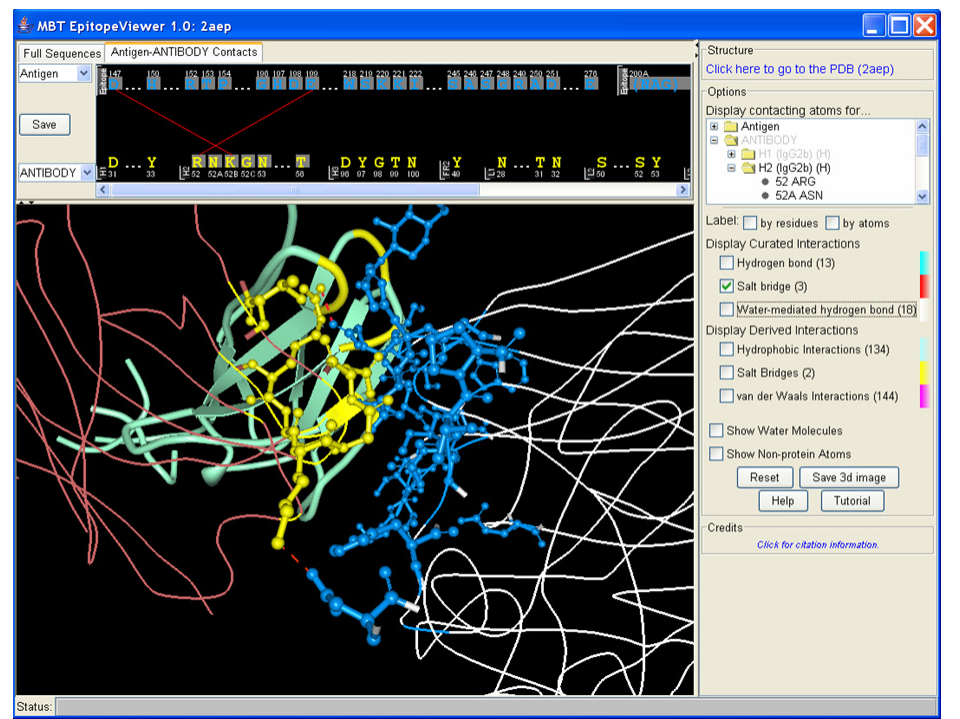
A screen-shot of the EpitopeViewer demonstrating salt bridge contacts between neuraminidase and CDR H2 of the Mem5 Fab. CDR H2 residues interacting with the epitope are shown in yellow and in all-atom representation.

The EpitopeViewer uses as input both PDB data in XML format and XML-files with curated data generated from the IEDB. The current version (1.0) of the tool has a control panel on the right hand side and four view windows: a 3D structure window; a tree window (window showing a hierarchical view of antigen and antibody on the right control panel); a sequence window ("Full Sequences" window in Fig. 1); and a contact window ("Antigen-Antibody Contacts" window in Fig. 2).

**Figure 3:**
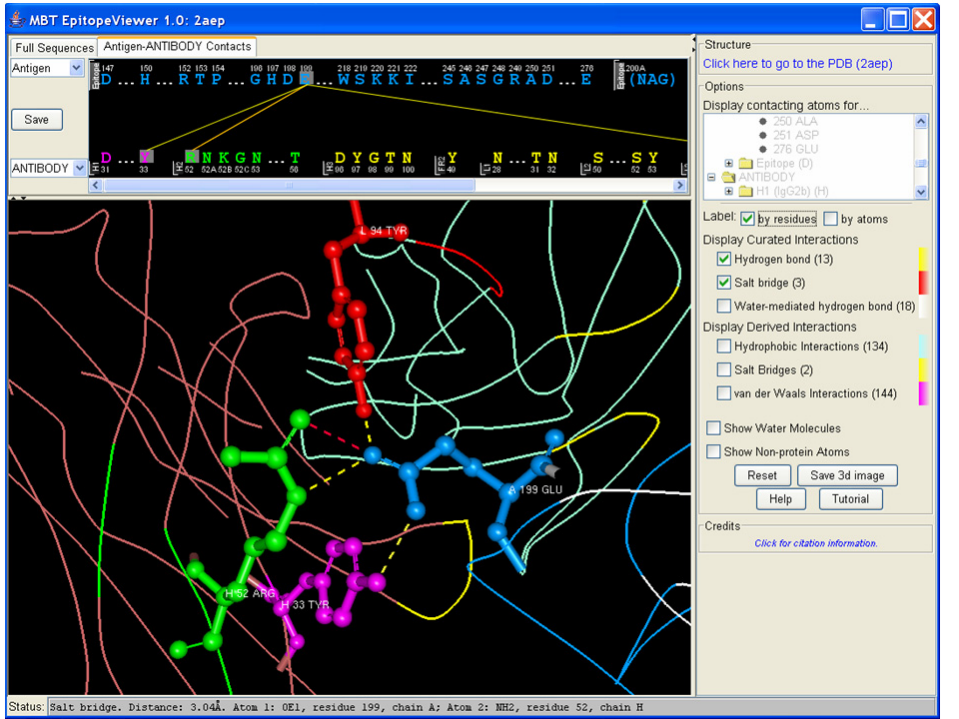
A screen-shot of the EpitopeViewer demonstrating electrostatic contacts between Glu199 neuraminidase residue (shown in blue) and antibody residues Tyr33 of CDR H1 (colored in magenta), Arg52 of CDR H2 (colored in green) and Tyr94 of CDR L3 (colored in red). Salt bridges and hydrogen bonds are shown by red and yellow dotted lines, respectively. Status of the "Status" bar corresponds to the mouse position showing the contact between Glu199 and Arg52 of CDR H2.

The *3D structure window *provides a renderable view of the structure of the immunological complex (Fig. 1), epitope and immune receptor residues interacting with epitope in an all-atom representation (Fig. 2) or contacts between epitope and receptor and their specific atomic interactions (Fig. 3). By default the EpitopeViewer draws protein chains of the antigen and receptor(s) as solid ribbons with each protein chain plotted in a different color. Epitope residues are colored in blue and immune receptor residues interacting with the epitope in yellow. Epitope residues which are not amino acids are plotted in all-atom representation by default. The 3D structure view can be saved in a variety of graphics formats using the "Save 3D Image" button on the right control panel of the viewer.

The *tree window *provides a hierarchical view of the components of a given molecule: chains, Complementarity-Determining Regions (CDRs ; where curated) and residues. The tree window allows the user to select particular components for further manipulation (e.g., labeling, coloring) and analysis of interactions.

The *sequence window *displays the primary sequences of the antigen and receptor protein chains. For antigens other than proteins (carbohydrates, DNA/RNA, etc.) the residue names are not displayed but rather marked by asterisks.

The *contact window *provides a 2D plot of interactions between epitope and receptor residues. Contacts are not shown by default; they are displayed using the control panel options by selecting particular type(s) of contacts for visualization (antigen, receptor or their particular chains, CDR(s) or residues) in the tree window. Selection can also be done in the 3D structure or sequence window by clicking on appropriate residues.

Color coding is preserved between all windows. The default colors for the epitope, antigen, immune receptor interacting residues and receptor chains have been chosen to satisfy color-blind users using the Vischeck software [23].

Curated data from the IEDB on immune receptor residues interacting with the epitope (labeled as paratope for antibodies) are displayed by default. In cases where the IEDB does not provide such curated information, the receptor residues interacting with the epitope are calculated by the EpitopeViewer based on salt bridges, van der Waals and hydrophobic inter-atom interactions. These interactions between epitope and immune receptor are calculated by the viewer on-the-fly and provided for both curated and calculated receptor residues interacting with the epitope. Salt bridges (or ion pairs) are identified for the charged side-chain atoms of Asp, Glu, Arg, Lys and His amino acids. It is assumed that negatively charged side-chain atoms of amino acids Asp (atoms OD2, OD1), Glu (atoms OE2, OE1) are capable of forming salt bridges with positively charged side-chain atoms of amino acids Arg (atoms NH1, NH2), Lys (atom NZ), and His (atom ND1, NE2) within a 4Å distance [24]. To determine van der Waals contact between two atoms, we use a distance cutoff equal to the sum of two atomic radii plus 0.5Å. For amino acids we use van der Waals radii as defined in the program NACCESS [25], for other atoms van der Waals radii were taken from Bondi [26]. In deriving hydrophobic interactions we assume that two atoms are involved in hydrophobic interaction if they are separated from each other by a distance ≤ 5.0 Å and belong to the main chain of any amino acid or side chain of hydrophobic amino acids (Ala, Val, Pro, Phe, Met, Leu, Ile, Trp, Tyr, Cys).

A user tutorial (web page in html-format) and help page (plain text in pop-up window) are included with the EpitopeViewer.

## Results and Discussion

Version 1.0 of the EpitopeViewer provides the following functionality:

• Visualization and rendering of the 3D structure of an immunological complex and sequences of epitope, antigen, antigen-specific receptor(s), and CDRs curated within the IEDB.

• 3D-visualization of curated interactions between epitope and receptor as well as derived van der Waals and hydrophobic interactions (calculated on-the-fly) including contacting residues, atoms and contact distances.

• 2D-visualization (plot) of inter-molecular inter-residue interactions.

• Coloring of selected residue(s), CDR(s), protein chains, type of contacts for epitope/receptor interaction and background.

• Labeling of residues and atoms.

• Coloring of non-protein atoms according to atom type.

• Optional presentation of water molecules and non-protein atoms.

• Direct access to details of the structure found in the RCSB PDB.

• An option to save images of the 3D structure and interaction plot in jpeg graphic format and resolution (pixel size).

This functionality is demonstrated and discussed in this section using as an example the influenza A virus epitope.

Figures 1, 2, 3 presents the epitope of Influenza A virus (strain A/Memphis/31/98 H3N2) neuraminidase recognized by the Fab fragment of the monoclonal antibody Mem5 and curated in the IEDB [IEDB:1000520] according to the structure of neuraminidase in complex with Mem5 Fab [PDB:2AEP] [27]. Figure 1 is a screen-shot of the EpitopeViewer showing the structure of the complex and sequences of antigen and antibody. The neuraminidase is shown in white, epitope in blue, antibody light chain in bluish-green, antibody heavy chain in reddish-brown and paratope in yellow. The carbohydrate attached to the Asn200 residue of the antigen forms part of the epitope. It is shown in an all-atom representation colored blue. Figure 2 is a screen-shot of the EpitopeViewer showing the salt bridges between epitope residues and CDR H2 of the antibody. Figure 3 shows a close-up view of contacts between neuraminidase residue Glu199 and Mem5 Fab. Glu199 is the most critical residue to the interaction since mutations at position 199 were found in antibody escape mutant viruses that failed to interact with the Mem5 antibody [27]. It can be seen that this residue makes extensive electrostatic interactions with Tyr33 of CDR H1, Arg52 of CDR H2 and Tyr94 of CDR L3.

The viewer has been tested on curated structures available in the IEDB. Epitopes inferred from 3D structures of antigens in complexes with immune receptors can be retrieved and displayed in the EpitopeViewer from the IEDB web-site [2] using a quick link 'Browse Records by 3D Structure'.

## Conclusion

The EpitopeViewer is a web-accessible tool for immediate use in the visualization and analysis of 3D structures of immunological epitopes and their interaction with immune receptors curated and available in the IEDB database. The EpitopeViewer has been developed in collaboration with immunologists to maximize usability. The tool was tested by immunologists participating at the IEDB Analysis Tool Jamboree in Bethesda, USA (November 4, 2005) and their feedback addressed. Additional testing and development was facilitated through close collaboration with scientists at the La Jolla Institute for Allergy and Immunology (LIAI). The EpitopeViewer has been tested on structural epitopes curated and available in the IEDB.

## Availability and requirements

Project name: EpitopeViewer

**Project home page: **

**Operating system(s): **Platform independent

**Programming language: **Java

**Other requirements: **Java 1.4.2 Run Time Environment or higher

**License: **Free for educational, research and non-profit purposes

**Any restrictions to use by non-academics: **Contact the University of California at San Diego's Technology Transfer Office (invent@ucsd.edu, 1-858-534-5815)

## List of abbreviations

IEDB – Immune Epitope Database and Analysis Resource

IEDB ID – Unique IEDB numerical identifier assigned by the system to each epitope in every reference contained in the IEDB.

PDB – Protein Data Bank

PDB ID – four-characters Protein Data Bank Identification for the structure

MHC – Major Histocompatibility Complex

CDR – Complementarity-Determining Region

MBT – Molecular Biology Toolkit

3D – three-dimensional

XML – eXtensible Markup Language

## Competing interests

The author(s) declare that they have no competing interests.

## Authors' contributions

JB programmed the tool and participated in the design of the tool.

PEB is the Principle Investigator of the project, conceived the idea for the underlying MBT software, participated in the coordination of the project, suggested the general functionality and revised the manuscript.

JVP conceptualized and designed the tool, coordinated the project, provided data and scientific objectives for the tool and drafted the manuscript.

All authors read and approved the final manuscript.
